# Periodontoid Pseudotumor in Tuberous Sclerosis Associated With Neck Diffuse Lipomatosis

**DOI:** 10.7759/cureus.32663

**Published:** 2022-12-18

**Authors:** Eduarda Pinto, Marcos Veiga, Amets Sagarribay, Carla Conceição

**Affiliations:** 1 Neuroradiology, Centro Hospitalar Universitário do Porto, Porto, PRT; 2 Neuroradiology, Centro Hospitalar Universitário de Lisboa Central, Lisbon, PRT; 3 Neurosurgery, Centro Hospitalar Universitário de Lisboa Central, Lisbon, PRT

**Keywords:** neuroradiology, phakomatoses, periodontoid pseudotumor, diffuse lipomatosis, tuberous sclerosis

## Abstract

Tuberous sclerosis (TS) is a genetic multisystem disorder associated with the development of benign tumors in many organs. Diffuse lipomatosis, which represents the overgrowth of fatty tissue in one part of the body, is a very rare finding reported in patients with tuberous sclerosis. We describe the case of a patient with diffuse lipomatosis in the right scapular, posterior cervical and perivertebral regions, associated with a space-occupying lesion adjacent to the odontoid process of C2 that appeared to be a pseudotumor, and discuss possible relation between these entities.

## Introduction

Tuberous sclerosis (TS) is a genetic multisystem disorder, caused by a mutation in one of two different genes, TSC1 and TSC2, that encode for different tumor suppressor proteins known as hamartin and tuberin, respectively. In many cases, it occurs as a sporadic mutation, but it can also be inherited as autosomal dominant. Alterations in the TSC1 and TSC2 genes lead to exaggerated activity of the mammalian target of rapamycin (mTOR) pathway, causing the development of benign tumors in many organs including the skin, brain, eyes, kidneys, lungs, and heart. This disorder is clinically variable from one person to another, depending on the organs affected. Nearly all patients have skin abnormalities including hypomelanotic macule and angiofibromas. The central nervous system (CNS) is frequently involved with cortical/subcortical tubers, subependymal nodules, and subependymal giant astrocytoma (SEGA). Other major abnormalities include kidney angiomyolipomas, lymphangioleiomyomatosis of the lung, heart rhabdomyoma, and retinal hamartomas [1,2]. Six patients were reported to have diffuse overgrowth of fatty tissue in one part of the body, defined as diffuse lipomatosis [3-6].

We describe the case of a patient with diffuse lipomatosis in the right scapular and cervical regions associated with an incidental finding on MRI of a space-occupying lesion at the atlanto-axial joint similar to a pseudotumor, a previously unreported association, to the best of our knowledge.

## Case presentation

A 16-year-old patient was initially observed at a medical appointment because of a slowly growing lesion in the right scapular region over 4 years, composed of fatty tissue, as assessed by soft tissue ultrasound. He was diagnosed with tuberous sclerosis, with a mutation in the TSC1 gene.

**Figure 1:**
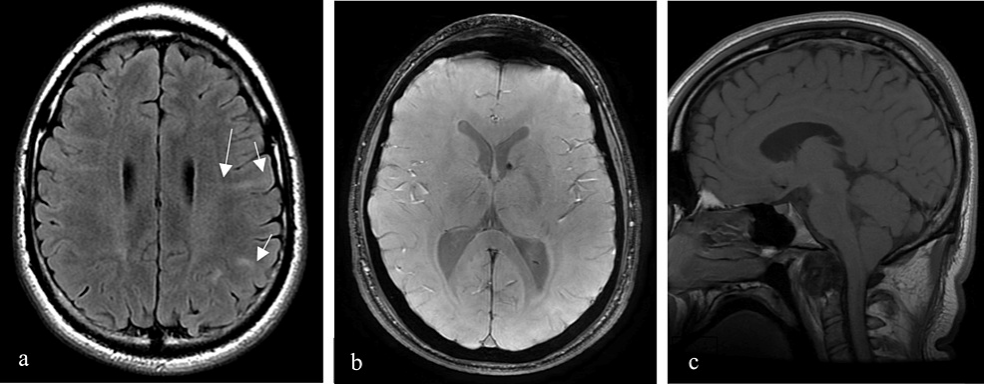
Brain MRI with axial T2FLAIR-weighted image (a), axial T2*-WI (b) and sagittal T1-WI (c). Cortical and subcortical tubers (short arrow) and radial migrating bands (long arrow) in both cerebral hemispheres are shown on axial T2FLAIR-WI (a). A small calcified subependymal hamartoma in the frontal horn of the left lateral ventricle is better depicted on axial T2*-WI (b). Sagittal T1-WI (c) shows a Chiari I malformation, with a short clivus and caudal descent of cerebellar tonsils, compressing the posterior surface of the medulla. It also reveals a space-occupying lesion at the atlanto-axial joint.

Brain magnetic resonance imaging (MRI) revealed cortical and subcortical tubers and radial migrating bands in both cerebral hemispheres and small subependymal hamartomas, related to tuberous sclerosis. A Chiari I malformation was also found, due to a short clivus, with a caudal descent of cerebellar tonsils, compressing the posterior surface of the medulla. As an incidental finding, a space-occupying lesion at the atlanto-axial joint was noted (Figure 1).

**Figure 2:**
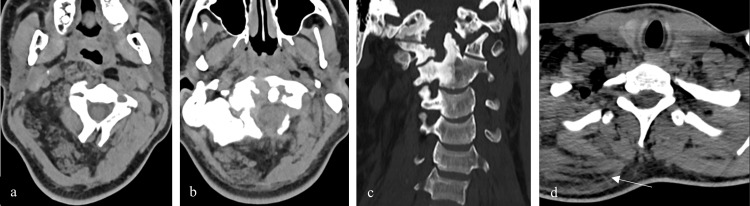
Cervical CT with axial images in soft window (a) (b) (d) and coronal reformation in bone window (c) demonstrates a diffuse lipomatous infiltration of the right suboccipital, posterior paravertebral, and prevertebral muscles, as well as the ipsilateral posterior cervical, retropharyngeal, and carotid spaces, with increased volume of these regions, deforming the posterior wall of nasopharynx and oropharynx (a). It reveals a periodontoid soft tissue mass (b), with remodeling of the right lateral mass of C2, the odontoid process and the anterior arch of C1 (c). The bone abnormalities were well delimited and exhibited cortical, subchondral and medullary sclerosis (c). Diffuse lipomatosis of the muscles and spaces of the right scapular region was also observed (arrow) (d).

**Figure 3:**
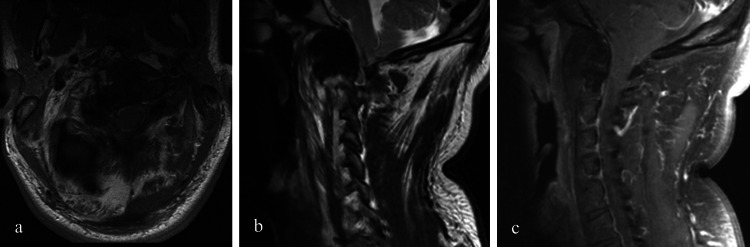
MRI of the cervical spine with axial T1-WI (a), sagittal T2-WI (b) and fat supressed contrast-enhanced sagittal T1-WI (c). Besides diffuse lipomatous infiltration, axial T1-WI (a) and sagittal T2-WI (b) show a hypointense mass at the atlanto-axial joint, extending into the anterior epidural space, but without compression of the brainstem or the spinal cord. No contrast enhancement is depicted on fat-suppressed contrast-enhanced sagittal T1-WI (c).

To better characterize this lesion, a cervical computed tomography (CT) was later performed. It demonstrated a diffuse lipomatous infiltration and enlargement of the right suboccipital, posterior paravertebral, and prevertebral muscles, and of the ipsilateral posterior cervical, submandibular, retropharyngeal, and carotid spaces. In addition, there was a periodontoid soft tissue mass, with bone remodeling of the right lateral mass of C2, the odontoid and the anterior arch of C1 and less pronounced remodeling of the clivus. These bone abnormalities were well delimited and exhibited cortical and subchondral sclerosis (Figure 2) which also extended to the ipsilateral C3 and C4 body and transverse process. Cervical spine MRI revealed a hypointense mass on T1 and T2-weighted images at the atlanto-axial joint, with no contrast enhancement. It extended into the anterior epidural space, decreasing the subarachnoid space, without compression of the brainstem or the spinal cord (Figure 3). The lesion remained stable compared to the brain MRI performed a few months earlier.

These imaging findings, namely the bone remodeling and sclerosis, and the stability of the changes through the different examinations suggested a process with slow growth and, therefore, a benign lesion. Its imaging features resembled those of a periodontoid pseudotumor. 

The patient had no symptoms related to this space-occupying lesion at the craniocervical junction, so a conservative expectant attitude was decided with clinical and imaging surveillance.

## Discussion

Diffuse lipomatosis in one part of the body is a very rare finding in patients with tuberous sclerosis and has been previously reported only in six patients. It is observed on the lower limbs in most cases, and in the subcutaneous lumbar region and the thoracic region around the lungs [3-6]. These previous reports are summarized in Table 1. Our patient had a diffuse lipomatous infiltration of the right neck spaces and the right paravertebral and prevertebral muscles, causing the tumefaction observed on physical examination at the right scapular region.

**Table 1:** Previous reports of Diffuse Lipomatosis in TS.

Publications	No. of patients	Age (years)	Diagnosis methods	Site
Klein et al. 1986 [6]	1	15	X-ray, biopsy/excision	Lower limb
Alcázar et al. 1998 [5]	1	37	CT scan, biopsy	Dorsal transthoracic
Mittal et al. 2017 [4]	1	24	MRI, fine needle aspiration	Lower limb
Ilyas et al. 2021 [3]	3	11	MRI, fine needle aspiration	Lower limb
		14	Ultrasound, MRI	Lower limb
		13	Ultrasound, MRI, biopsy/excision	Lower limb

We hypothesized that this diffuse lipomatous infiltration of the muscles and neck spaces led to an atlanto-axial joint hypermobility with the development of a presumed periodontoid pseudotumor.

Periodontoid pseudotumor reflects a non-neoplastic soft tissue mass adjacent to the odontoid process. It is caused by various mechanisms and etiologies such as rheumatoid arthritis, trauma, atlantoaxial hypermobility compensating for subaxial ankylosis, and deposition diseases (like calcium pyrophosphate). Some cases are idiopathic, but the underlying mechanism is believed to be a chronic subluxation of the atlanto-axial joint. The histology of the mass mainly reveals connective tissue and cartilage [7]. Supporting this hypothesis, previous surgery reports with posterior decompression and fusion led to a progressive spontaneous regression of the pseudotumor [7].

Though we do not have histologic confirmation in our case, we postulate that a periodontoid pseudotumor is the most plausible diagnosis, since it is a slowly growing extraosseous mass located around the odontoid process causing bone remodeling and sclerosis. The diffuse lipomatosis of the right neck spaces and muscles could explain an atlanto-axial subluxation and the development of an ipsilateral pseudotumor.

## Conclusions

Diffuse lipomatosis in one part of the body is a very rare finding in patients with tuberous sclerosis. Our patient was initially observed because of a slowly growing tumefaction in the right scapular region over 4 years. The first follow-up brain MRI of tuberous sclerosis revealed an incidental space-occupying lesion at the anterior atlanto-axial joint, the characteristics of which were of a periodontoid pseudotumor on both cervical CT and MRI. These imaging studies also revealed diffuse lipomatosis in the right cervical spaces and ipsilateral perivertebral muscles, leading to a probable association between these two entities.
